# Lipopolysaccharide stimulation test on cultured PBMCs assists the discrimination of cryopyrin-associated periodic syndrome from systemic juvenile idiopathic arthritis

**DOI:** 10.1038/s41598-021-91354-5

**Published:** 2021-06-07

**Authors:** Chao-Yi Wu, Wen-Lang Fan, Ying-Ming Chiu, Huang-Yu Yang, Wen-I. Lee, Jing-Long Huang

**Affiliations:** 1 Division of Allergy, Asthma, and Rheumatology, Department of Pediatrics, Chang Gung Memorial Hospital, No.5 Fu-Hsing St., Taoyuan, Taiwan, ROC; 2 College of Medicine, Chang Gung University, Taoyuan, Taiwan, ROC; 3 Genomic Medicine Core Laboratory, Chang Gung Memorial Hospital, Taoyuan, Taiwan, ROC; 4 Department of Allergy, Immunology and Rheumatology, Tungs’ Taichung Metroharbor Hospital, Taichung, Taiwan, ROC; 5 Department of Nephrology, Chang Gung Memorial Hospital, Taoyuan, Taiwan, ROC; 6 Department of Pediatrics, New Taipei Municipal TuCheng Hospital, New Taipei city, Taiwan, ROC

**Keywords:** Paediatric research, Immunological disorders

## Abstract

Systemic juvenile idiopathic arthritis (sJIA) and cryopyrin-associated periodic syndrome (CAPS) share many common manifestations. We aim to identify an applicable method to assist disease discrimination. Inflammatory cytokines were measured in the plasma of patients with CAPS, sJIA with persistent disease course and healthy controls. Supernatants collected from non-stimulated peripheral blood mononuclear cells (PBMCs) and those undergone inflammasome stimulation tests utilizing lipopolysaccharide (LPS) with and without adenosine triphosphate (ATP) were investigated. Inflammatory cytokines in patient plasma fail to differentiate sJIA from CAPS. PBMCs from sJIA secrets higher amount of IL-1β and IL-18 while CAPS PBMCs produces more caspase-1 without stimulation. IL-1β, IL-18, and caspase-1 were significantly elevated among CAPS PBMCs (all *p* < 0.05) upon LPS stimulation, but not when additional ATPs were provided. Levels of cytokines and PBMC responses to the stimulation assays were similar among all sJIA patients regardless of their history of macrophage activation syndrome. Unstimulated PBMC activities and the LPS inflammasome stimulation assay without exogenic ATPs can assist the differentiation of CAPS from sJIA with persistent disease course.

## Introduction

Cryopyrin-associated periodic syndrome (CAPS) is a rare autoinflammatory syndrome characterized by overproduction of interleukin (IL)-1β, resulting from dysregulated NOD-like receptor family pyrin domain containing 3 (NLRP3) inflammasome activity^1,2^. CAPS-related inflammation can cause fever, fatigue, skin irritation and musculoskeletal symptoms at the early stages^3^. Persisted uncontrolled inflammation, moreover, can lead to serious organ damages, including sensorineural hearing defect, renal amyloidosis, skeletal deformities and cognitive impairments^1,4^. To assist early diagnosis, provisional clinical classification criteria for autoinflammatory diseases and diagnostic criteria for CAPS have been introduced^5^. However, due to its board clinical phenotypes^6^, making a diagnosis of CAPS in clinic can be challenging^7^. In fact, the diagnosis of CAPS usually requires genetic evidence of NLRP3/cold-induced autoinflammatory syndrome 1 (CIAS1) germline gene mutations^8^, while mutations in NOD-like receptor family pyrin domain containing 12 (NLRP12) and somatic mosaicism of NLRP3 were also reported^9,10^.

Systemic juvenile idiopathic arthritis (sJIA), likewise, is a clinically diagnosed rheumatic disorder marked by over-activation of the innate immune system. Similar to CAPS, it is one of the most prevalent diseases within the scope of autoinflammation with heterogenic systemic manifestations^2,11,12^. While the course of sJIA may be monophasic or polycyclic, around half of the sJIA cases display a persistent disease course of relapsing high fever associating arthritis, skin rash, lymphadenopathy and serositis^13^. Additionally, around 6.7%–13% of the patients with sJIA experienced a potentially fatal condition, macrophage activation syndrome (MAS), portrayed by persistent fever, pan-cytopenia, liver abnormalities, lymphadenopathy, coagulopathy and neurological involvement^14,15^. Overlapping clinical manifestations, mutual hyper-active innate responses as well as common serum and cellular biomarkers of CAPS and sJIA, particularly those with polycyclic and persistent disease course, can sometimes cause confusion for clinicians^3,16^. While the advance of molecular genetic testing was believed to provide strong support to the diagnosis, in a large French study covering 821 cases suspicious with CAPS, only 16% of the cases were identified with *NLRP3* mutations^10^. In addition, with more than 200 NLRP3 sequence variants registered in the Infevers database associating with CAPS, the pathogenic role of half of the variants are of uncertain significance^17^.

Many shared gene transcripts have been identified in patients with sJIA and CAPS^18,19^. Considerable body of works, additionally, have discussed about the gene expression profiling, cellular markers and serum biomarkers among the two diseases^16^. Limited data, however, were available directly comparing the markers between the two. Hence, it is not known whether or not these markers may be applicable in clinical settings to assist the disease discrimination. To investigate whether inflammatory markers and inflammasome stimulation tests can assist the differentiation of CAPS from persistent sJIA, we evaluated plasma inflammatory cytokine levels as well as the production of inflammatory cytokines produced by the peripheral blood mononuclear cells (PBMCs) upon lipopolysaccharide (LPS) with and without exogenic adenosine triphosphates (ATPs) stimulation in cases with CAPS, persistent sJIA and healthy controls. Moreover, we also evaluated the cytokines and inflammasome activities in sJIA patients with and without a history of MAS. Our data suggested that LPS inflammasome stimulation test without exogenic ATPs, and not plasma inflammatory cytokines perform better in differentiating between the two.

## Material and methods

### Study population

Eleven patients who fulfilled the 2017 diagnostic criteria for CAPS^5^ in a pediatric rheumatology clinic of a tertiary medical center in Taiwan were invited to participate the study at time of diagnosis or at time of treatment initiation with active diseases. Twelve cases diagnosed with sJIA according to the international league of associations for rheumatology classification^11^, with active disease for more than 24 months' duration and still remain in an active disease status were enrolled. Additionally, 11 healthy individuals were also invited as controls. Cases with CAPS have raised acute phase reactants and experienced at least 2 of the following clinical manifestations: recurrent fever, urticarial-like skin rashes, cold or stress triggered attacks, sensorineural hearing deficit, chronic aseptic meningitis, skeletomuscular symptoms or abnormalities, regardless of their family history. Patients diagnosed with sJIA suffered from arthritis and symptoms of systemic inflammation such as fever, skin rash and serositis before the age of 16. MAS was diagnosed according to the 2016 classification criteria for MAS complicating sJIA^20^. All subjects were excluded for infectious and oncological causes. Written informed consents were collected from all the participated subjects and/or their legal guardian. The research was in compliance with the Declaration of Helsinki and was approved by the Chang Gung Memorial Hospital Institutional Review Board (IRB No.: 201802287A3).

### Genetic analysis

*NLRP3/CIAS1* gene [NCBI RefSeqGene NC_000001.9] sequencing covering all 9 exomes were analyzed by sanger sequencing using the standard protocol with proper negative and positive controls in each polymerase chain reaction (PCR) test. Specifically, DNA fragments were amplified by ABI PCR machine with designated primers and FastStart Taq DNA polymerase (Roche, Basel, Switzerland). PCR was validated with gel electrophoresis, and the final products were purified with FavorPrep GEL/PCR Purification Kit (Favorgen, Taiwan) according to the manufacturer's protocol. Sequencing reaction was performed with Big Dye Terminator v. 3.1 Ready Reaction Cycle Sequencing kit (Applied Biosystems, Warrington, UK). The electrophoretic profiles of *NLRP3* sequences were analyzed on the ABI 3500 Genetic Analyzer.

For those negative of *NLRP3/CIAS1* missense mutations, whole exomes sequencing were performed to expand the coverage of other autoinflammatory disease related gene mutations. In detail, exome capture were performed using the Agilent SureSelect Human All Exon Kit V6 (Agilent Technologies) and massively parallel sequencing were carry out using the NovaSeq 6000 (Illumina, San Diego, CA). Raw image analyses and base calling were performed using Illumina’s Pipeline with default parameters. Sequence data were aligned to the reference human genome (hs37d5) using the Burrows-Wheeler Aligner^21^, and duplicate reads were removed using Picard tools. We use the Genome Analysis ToolKit (GATK 4.1.2) Haplotype Caller for variant calling of SNVs and short (< 50 bp) indels^22^. Annovar were utilized to catalogue the detected variations^23^. Then, we filtered variations with a homopolymer length > 6 (and synonymous substitutions) or that were common (> 1%) in dbSNP150 (http://www.ncbi.nlm.nih.gov/projects/SNP/), HapMap, the 1000 Genomes Project (http://www.1000genomes.org), the Exome Aggregation Consortium database and the Genome Aggregation Database (GnomAD, https://gnomad.broadinstitute.org). Integrated genome viewer was used to visualize the reads for manual checking.

### Protein measurements

Levels of IL-1β, IL-6, IL-18, tumor necrosis factor alpha (TNF-α) and caspase-1 p20 within frozen plasma and culture soup were measured by sandwich enzyme-linked immunosorbent assay reagent kits (DY201-05, DY206-05, DY318-05, DY210-05 and DCA100, respectively) obtained from R&D system (Minneapolis, MN, USA). Serum amyloid A (SAA) was measured by sandwich enzyme-linked immunosorbent assay reagent kits (KA4865) obtained from Abnova (Taipei, Taiwan). The assays were performed according to the manufacturer’s instructions. Appropriate recombinant human protein were used to establish the standard curve for each assay, respectively.

### Inflammasome stimulation test

After isolating human peripheral blood mononuclear cells via Ficoll-Paque gradient centrifugation, cells were counted and seeded onto 96-well plates at a concentration of 3 × 10^6^ cells/ml in culture medium (RPMI medium with 10% heat-inactivated autoserum, 100 U/ml penicillin and 2 mM L-glutamine). The cells were either stimulated with LPS 1 µg/ml (Sigma) for 1 or 4 h, or primed with LPS 1 µg/ml for 1 h then stimulated with ATP 2 mM (Sigma) for another 1 or 4 h after cell seeding. Supernatants collected at 1 h after PBMC seeding without stimulation were used as non-stimulation controls. The culture supernatants were collected for the analysis of IL-1β, IL-18 and caspase-1 secretion with and without stimulations. All tests were performed in duplicates.

### Statistical analysis

Continuous data were summarized as means ± SDs and compared by unpaired t-test. Nonparametric Mann–Whitney sum rank *U* test and Kruskal–Wallis (χ^2^) test were used for the between-group comparison. The level of significance is determined to be at 0.05. Statistical analyses and graphic presentation were carried out using Prism 6.01 software (GraphPad Software, Inc., San Diego, California).

### Ethics approval and consent to participate

This study was approved of by the Institutional Review Board of the Chang Gung Memorial Hospital (IRB No.: 201802287A3).

## Results

### Clinical characteristics and inflammatory markers in cases with CAPS and sJIA

The average age of onset is 6.2 ± 5.7 years among cases with CAPS and 6.7 ± 4.8 for cases with persistent sJIA. Age at time of recruitment was 23.8 ± 18.0, 13.6 ± 7.8 and 18.3 ± 9.3 for CAPS, sJIA and healthy controls, respectively. The gender distribution, levels of acute phase reactants, laboratory profiles as well as the treatment regimens at time of sampling were summarized in Table 1. Three of the cases (25%) suffered from persistent sJIA experienced at least one MAS episodes after the diagnosis of sJIA.

**Table 1:** Characteristics of the study cases.

	CAPS	sJIA	Healthy controls
Case number	11	12	11
Age of disease onset (year)	6.2 ± 5.7	6.7 ± 4.8	–
Age (year)	23.8 ± 18.0	13.6 ± 7.8	18.3 ± 9.3
Gender (M/F)	2/9	6/6	5/6
Family history (%)	8 (72.7%)	0	0
NLRP3 variants	A439V: n = 5 E457D: n = 1 V70M: n = 1	–	–
Phenotypes (MWS/FCAS)	3/8	–	–
MAS (%)	0	3 (25%)	–
WBC (/uL)	10,118.2 ± 3629.3	14,616.7 ± 8531.6	–
Hb (g/dL)	13.4 ± 1.3	10.3 ± 3.6	–
platelet (10^3^/uL)	290.0 ± 62.4	392.4 ± 189.2	–
CRP (mg/dL)	12.9 ± 14.9	28.3 ± 21.4	–
ESR (mm/h)	25.2 ± 9.5	36.7 ± 33.9	–
IgG (mg/dL)	1118.5 ± 182.6	1552.6 ± 803.7	–
ANA (%)	2 (18.2%)	1 (8.3%)	–
Anti-IL-6R (%)	0	9 (75%)	–
Anti-IL-1β (%)	1 (9.1%)	0	–
Anti-TNF⍺ (%)	0	0	–
Steroid (%)	1 (9.1%)	4 (33.3%)	–
NSAID (%)	0	2 (16.7%)	–
MTX (%)	0	9 (8.3%)	–

Abbreviations: CAPS-cryopyrin-associated periodic syndrome; sJIA-systemic juvenile idiopathic arthritis; NLRP3-NOD-like receptor family, pryin domain containing 3; MWS-Muckle–Wells syndrome; FCAS-familial cold autoinflammatory syndrome; MAS-macrophage activation syndrome; WBC-white blood cells; Hb-hemoglobulin; CRP-c-reactive protein; ESR-erythrocyte sedimentation rate; IgG-immunoglobulin G; ANA-antinuclear antibodies; IL-6R-interleukin 6 receptor; IL-1β-interleukin 1β; TNF⍺-tumor necrosis factor ⍺; NSAID-nonsteroidal anti-inflammatory drug; MTX-methotrexate.

Among the 11 patients with CAPS, three cases presented with a phenotype compatible with Muckle–Wells syndrome (MWS) while the other 8 experienced recurrent urticarial-like skin rashes especially upon cold or stress triggers. Five patients from 2 distinct family were found to harbor heterozygous c.1316 C > T (p.A439V) NLRP3 variants, which has been confirmed pathogenic according to the Infever database^17^. Additionally, two independent cases carrying heterozygous NLRP3 variants with undetermined clinical significance c.210 G > A (p.V70M) and c.1371 G > T (p.E457D) were also detached. The minor allele frequency for c.210 G > A and c.1371 G > T is 0.000741 and 0.00008 in the Trans-Omics for Precision Medicine program and 0.0077 and 0.0013 among the East Asians in the GnomAD database, respectively. Somatic mosaicism of NLRP3 were not identified using the sequencing technique applied. Cases diagnosed with persistent sJIA were negative for known genetic mutations associating autoinflammatory diseases. Clinical manifestations and relevant comorbidities of the patients diagnosed with CAPS were summarized in Supplementary Table 1.

### Differences in the level of inflammatory cytokines between cases with CAPS and sJIA

**Figure 1:**
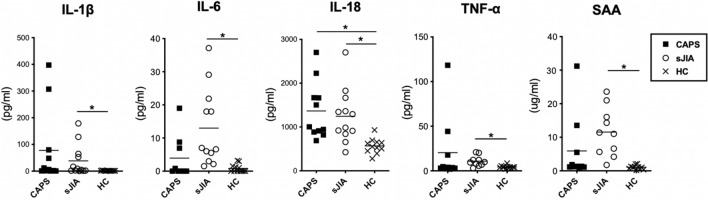
Levels of inflammatory cytokines and serum amyloid A in patients’ plasma. Levels of inflammatory cytokines IL-1β, IL-6, IL-18, TNF-α and SAA were measured in cases with CAPS, sJIA and healthy controls. While the cytokines were globally elevated among the sJIA patients (*p* < 0.05), the level of IL-6 within the sJIA patient plasma is increased as compared to those with CAPS with borderline significance (*p* = 0.051). Data are presented with dot plot.* indicates *p* < 0.05. Abbreviations: CAPS- cryopyrin-associated periodic syndrome; sJIA-systemic juvenile idiopathic arthritis; HC- healthy controls; IL-1β- interleukin 1β; IL-6- interleukin 6; IL-18- interleukin 18; TNF⍺- tumor necrosis factor ⍺; SAA- serum amyloid A.

CAPS and sJIA were both systemic inflammatory diseases with overactivated innate immune responses. To investigate whether there are differences in the plasma profile of inflammatory cytokines, levels of IL-1β, IL-6, IL-18, TNF-α and SAA were examined from patients’ plasma. As shown in Fig. 1, while an elevation in the level of IL-18 was observed in patients with CAPS when compared to the healthy controls (1366.1 ± 196.2 vs. 568.8 ± 171.9; *p* = 0.0008), the differences in levels of plasma IL-6 between CAPS and persistent sJIA were borderline significant (*p* = 0.051). Additionally, patients with sJIA have higher levels of inflammatory cytokines in all parameters tested when compared to healthy controls (38.6 ± 58.68 vs. 1.5 ± 0.7, 13.0 ± 11.7 vs. 0.8 ± 1.2, 1244.0 ± 612.8 vs. 568.8 ± 171.9, 10.8 ± 5.8 vs. 4.4 ± 1.6 and 11.6 ± 7.2 vs. 1.1 ± 0.6 for IL-1β, IL-6, IL-18, TNF-α and SAA; *p* = 0.04, 0.002, 0.002, 0.001 and 0.001 respectively).

### Differences in inflammasome stimulation test between cases with CAPS and sJIA

**Figure 2:**
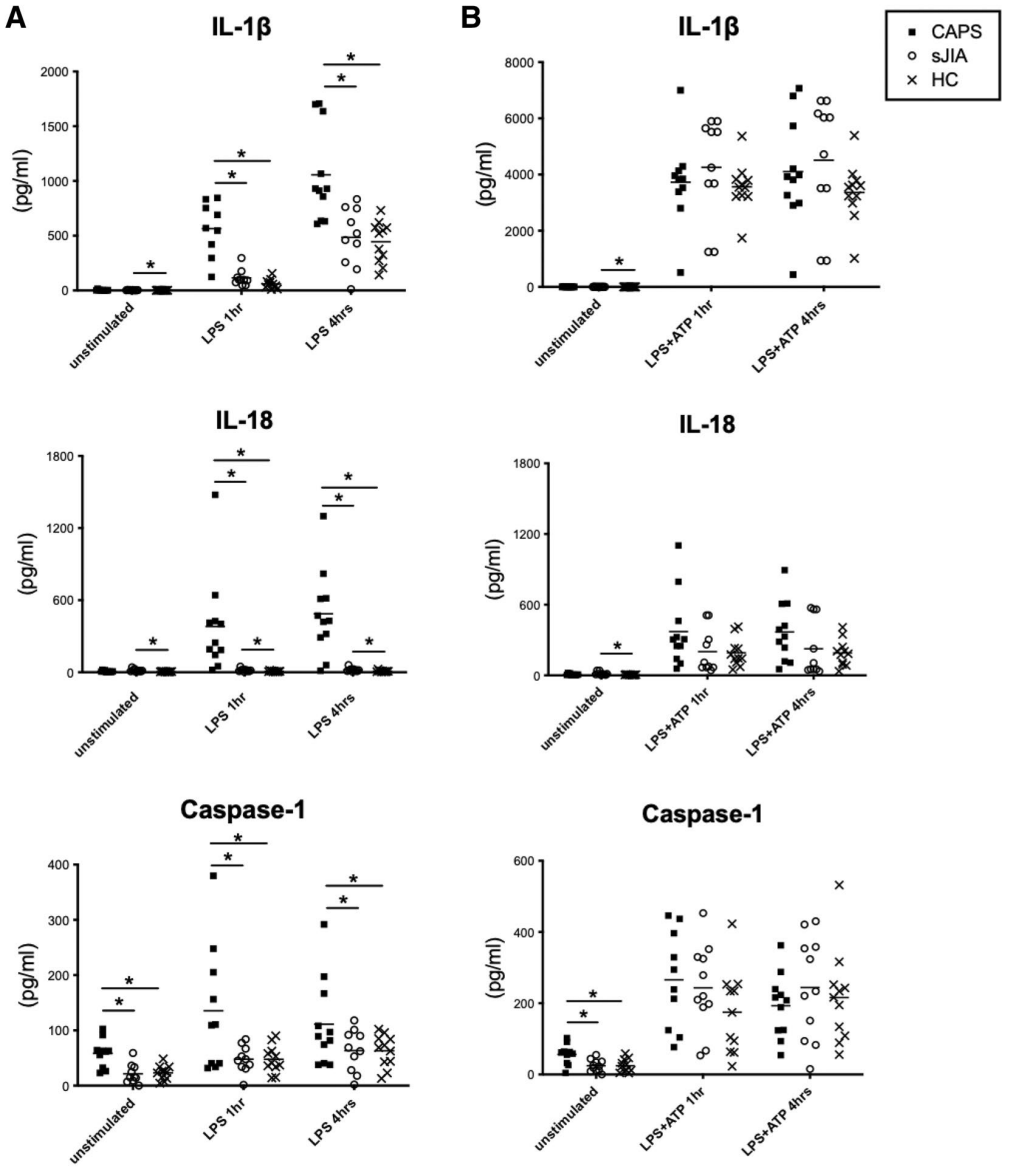
Secretion of inflammatory cytokines upon in vitro stimulation test. Secretion of IL-1β, IL-18 and caspase-1 differentiates between patients with CAPS, sJIA and healthy controls. IL-1β, IL-18 and caspase-1 secretion were measured in the supernatant of PBMCs isolated from patients with CAPS, sJIA and control donors with medium only and 1 and 4 h after A) LPS stimulation, and B) LPS-primed ATP stimulation regimen. Data are shown as dot plat with means. * indicates *p* < 0.05. Abbreviations: CAPS- cryopyrin-associated periodic syndrome; sJIA-systemic juvenile idiopathic arthritis; HC- healthy controls; IL-1β- interleukin 1β; IL-18- interleukin 18; LPS- lipopolysaccharide; ATP- adenosine triphosphate; PBMC- peripheral blood mononuclear cells.

Considering the critical role of NLRP3 inflammasome in CAPS and its possible effect in sJIA^8,24,25^, we evaluated inflammatory cytokines production from patient PBMCs with and without NLRP3 stimuli using plain culture soup alone, LPS and LPS-primed ATP regimen. Shown in Fig. 2A, significant increase in the levels of IL-1β, IL-18 and caspase 1 were detected in the soup of PBMCs isolated from patients with CAPS at 1 h and 4 h upon LPS stimuli in comparison to those isolated from sJIA and healthy controls (all *p* < 0.05). While higher levels of IL-18 were produced by sJIA PBMCs consistently regardless of LPS stimuli when compared to those healthy controls (*p* = 0.01, 0.03, 0.05, without and 1 and 4 h of stimulation, respectively), the production of IL-1β and caspase-1 did not significantly increase in sJIA PBMCs as compared to healthy controls upon LPS stimulation (all *p* > 0.05). Interestingly, caspase-1 production is increased in the CAPS PBMCs when compared to those isolated from sJIA and healthy controls before additional stimulants were applied (all *p* < 0.05). Moreover, the levels of IL-1β were also slightly higher in the sJIA PBMCs as compared to the healthy controls before stimulation (*p* = 0.03).

Extracellular ATP is a potent activator for NLRP3 inflammasome^26^. When PBMCs were provided with additional ATPs followed by LPS priming, the levels of caspase 1 and IL-1β increased by 2 to 4 folds universally, as shown in Fig. 2B. However, as the production of inflammatory cytokines were significantly increased, the differences in the levels of IL-1β, IL-18 and caspase 1 between those with or without diseases may be limited. Indeed, no significant differences in the inflammatory mediators were detached in the culture soup of PMBCs isolated from patient diagnosed with CAPS, sJIA and healthy controls (all *p* > 0.05).

### Differences in plasma cytokine levels and inflammasome responses in sJIA patients with and without a propensity of MAS

**Figure 3:**
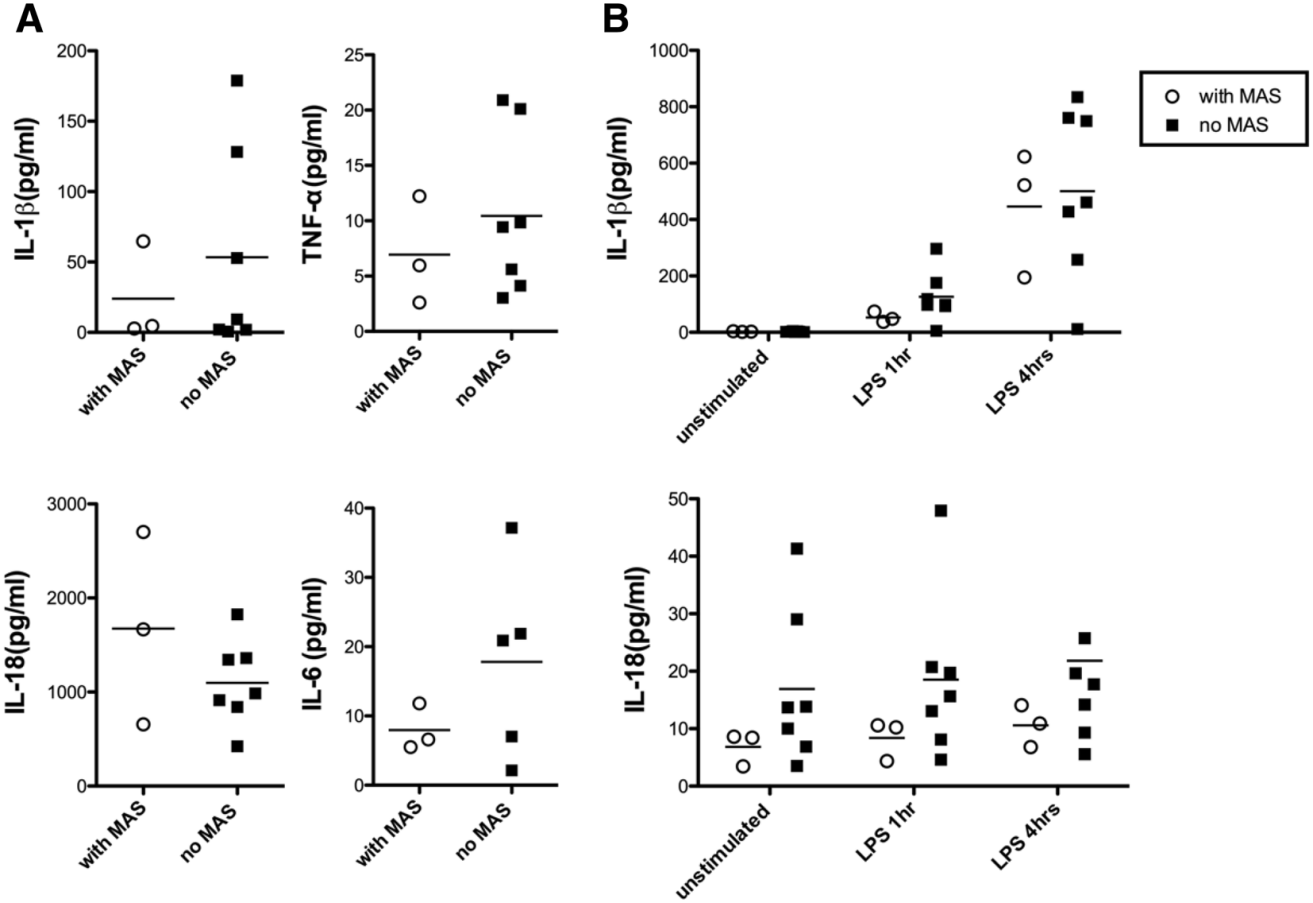
Plasma cytokine levels and PBMC responses to in vitro stimulations test among sJIA patients with and without a history of MAS. Levels of plasma cytokines and responses to in vitro stimulations tests are comparable among sJIA patients regardless of the propensity of MAS. (**A**) Levels of IL-1β, IL-6, IL-18 and TNF-α were measured and compared between sJIA cases with and without a history of MAS. (**B**) IL-1β and IL-18 secretion were measured in the supernatant of PBMCs isolated from patients with sJIA with medium only and 1 and 4 h after LPS stimulation. Results between those with and without a history of MAS were compared. Data are presented with dot plot with means. Abbreviations: sJIA-systemic juvenile idiopathic arthritis; MAS- macrophage activation syndrome; IL-1β- interleukin 1β; IL-6- interleukin 6; IL-18- interleukin 18; TNF⍺- tumor necrosis factor ⍺; LPS- lipopolysaccharide; ATP- adenosine triphosphate.

To investigate whether the propensity of MAS correlated with inflammasome responses or levels of plasma cytokines in cases with sJIA, we compared the inflammatory mediators acquired from 3 sJIA who experienced at least one episodes of MAS during follow up to those without. As shown in Fig. 3A, no significant differences were observed in the levels of plasma IL-1β, IL-6, IL-18 or TNF-α between sJIA patients regardless of the history of MAS (all *p* > 0.05). Moreover, the production of IL-1β and IL-18 in PBMCs upon LPS stimuli were also similar (Fig. 3B).

## Discussions

In the present study, we found that while inflammatory cytokines were generally elevated in persistent sJIA patients’ plasma as compared to the healthy controls, differences between CAPS and sJIA patients were not statistically significance. Moreover, the production of IL-1β, IL-18, and caspase-1 in the inflammasome activation assay were significantly increased among CAPS patients in comparison with sJIA patients or healthy individuals 1 or 4 h following LPS stimulation. This reaction, however, is masked by the universal elevation of inflammatory cytokines when exogenic ATPs were added. Finally, our data suggested that the levels of plasma cytokines and PBMC responses to the LPS stimulation assays were similar among all sJIA patients regardless of their propensity of MAS.

Nirmala et al. recently reviewed the similarities and differences of the protein, cellular, mRNA and DNA markers among patients suffering from sJIA and CAPS^16^. While inflammatory markers including IL-6, IL-18, S100A8/A9 and S100A12 were noted to elevate under both conditions^27–31^, limited reports have yet directly compared CAPS and sJIA for their expression of biomarkers. In a case series reported by Ohnishi et al., one patient with active JIA presented with higher levels of serum IL-6 and IL-18 in comparison with those with CAPS and healthy controls^3^. S100A8 and S100A9 have also been found to increase significantly among cases with sJIA, particularly during active status, when compared to patients with neonatal onset multisystem inflammatory disease (NOMID) and other inflammatory disorders^30^. As the activity status of sJIA largely influenced the level of serum cytokines^30,32^, sampling sJIA patients during chronic active phases, our data on inflammatory cytokines show no differences between cases suffered from CAPS and sJIA (Fig. 1). Thus, while our data echoed previous reports on a global increase of proinflammatory cytokines in cases with sJIA and CAPS^16^, no single cytokine along is capable of assisting the differentiation of CAPS from cases with persistent sJIA.

Physiologically, NLRP3 cryopyrin, an intracellular sensor, forms inflammasome complex associating pro-caspase-1 and ASC proteins upon encountering its triggers^26^. The formation of inflammasome then catalyzes pro-IL-1β and pro-IL-18, produce upon the activation of nuclear factor kappa B (NF-κB) signaling, to their mature form and potently drives further inflammation^26,33^. According to our result, no spontaneous secretion of IL-1β and IL-18, but caspase 1 was noted in CAPS PBMCs before any stimulation was applied (Fig. 2A,B). This may be explained by the necessity of NF-κB signaling for the transcription of pro-IL-1β and pro-IL-18, before they can be catalyzed by the over-active NLRP3 inflammasomes^34,35^. On the contrary, caspase-1 is a direct interaction partner for the NLRP3 inflammasome and is critical for its proteolytic activity in processing the precursors of various inflammatory cytokines^33^. Even without additional triggers, altered NLRP3 proteins oligomerized with pro-caspase-1 and displayed a higher basal caspase-1 activity as compared to healthy controls^36^. Nonetheless, while several reports also demonstrated the requirement of NF-κB signaling for the production of IL-1β and IL-18 in in vitro stimulation assays^3,37,38^, others reported high unstimulated cytokine production, including IL-6, IL-18, TNF, interferon (IFN)-γ and IL-12p70 in PBMCs isolated from patients suffered from CAPS^39,40^. These differences may be influenced by the disease status and severity at time of sampling. Furthermore, PBMCs from sJIA secreted higher amount of IL-1β and IL-18 before any additional stimulants were provided (Fig. 2A,B). Although the exact mechanism is unknown, genes involved in innate immune reaction, such as IL-1, IL-18 and toll like receptor (TLR) signaling pathways have been found to upregulate in cases with sJIA^41^.

The most pronounced difference between CAPS and persistent sJIA in the present study is perhaps the reaction of PBMCs upon LPS stimulation (Fig. 2A). While different mutations and its capacity to auto-activate the inflammasome can lead to large variability in the responses to stimulation^37^, IL-1β, IL-18, and caspase-1 were significantly elevated among CAPS PBMCs (all *p* < 0.05) upon LPS stimulation, but not when additional ATPs were provided. Experiments carry out in MWS murine models revealed a lower threshold and enhanced production of IL-1β and IL-18^42,43^. Likewise, enhanced IL-1β, IL-18 and caspase-1 release have been shown in CAPS PMBCs in comparison to control PBMCs^34,37^. Mutations in NLRP3 is believed to drive inflammation in cases with CAPS. Although polymorphisms in NLRP3 have also been reported to associate with sJIA^25^, it did not lead to an altered inflammatory response upon TLR stimulation. Additionally, while Reiber et al. observed a significant increase of mature IL-1β, IL-18, and caspase-1 in culture supernatants 4 h following LPS stimulation^37^, our result suggested that the differences may be detected at an earlier time point. The rapid and colossal secretion of inflammatory cytokines upon LPS stimulation is a reflection of the efficient processing of pro-cytokines in CAPS with constitutive active NLRP3 inflammasomes^1,2,4^. Moreover, priming the PBMCs with LPS followed by addition ATP in the samples showed global elevation in the inflammasome related products regardless of the underlining diseases (Fig. 2B). This was distinct from Reiber’s report, perhaps because their PBMCs received dual stimulation with ATPs and LPS simultaneously at a dose 10 times higher^37^. ATP is a potent activator for NLRP3 inflammasome^26^. The exogenic ATPs provided in the present protocol somehow activated NLRP3 inflammasomes to a comparable degree as they were in CAPS and masked the differences when LPS stimulation was provided. Together, our data suggested that LPS stimulation test is better than LPS and ATP together in identifying CAPS from patients with persistent sJIA and healthy controls.

Marked increase of proinflammatory cytokines including IL-1, IL-6, IL-18, TNFα, IFNγ, ferritin and ST2 during MAS attacks portrayed a significant systemic inflammation in patients with various rheumatic diseases, particularly sJIA^28,44–47^. Recently, Canna et al. reported that gain of function mutations in *NLRC4* can display a MAS like clinical presentation^48^. In addition, repetitive TLR stimulation via administration of CpG has been shown to induce a MAS-like features in mice^49^. Even though infections and inflammasome dysregulation have been considered as possible triggers for MAS^44,45^, the discrimination of immune responses between sJIA from CAPS with LPS stimulation tests in not jeopardized by the tendency for MAS in the present study with limited cases. Although further study may be required to confirm this observation, the similarity in the level of inflammatory cytokines and PBMC responses to LPS suggested that the induction of MAS in patients with sJIA may less likely result from NLRP3 inflammasome dysregulation.

The present study is limited by its few case numbers and only CAPS patients with the clinical phenotype of familial cold autoinflammatory syndrome (FCAS) and MWS were studied. It would be worthwhile to also examine patients with NOMID phenotype since the disease activity and phenotype have also been implied with altered inflammasome responses^39^. Moreover, considering the association of aging and inflammasome activity^50^, the imperfectly age-matched healthy controls can also result in bias in the present study. Finally, the in vitro functional diagnostic tests were performed in CAPS and sJIA patients under medical treatment with a relatively long disease duration. Although in clinical settings, the consideration of rare diseases and genetic testing are usually reserved for those who suffered from persisted disease course or those who respond poorly to standard treatments, future validation of the results with treatment-naïve patients before being correctly diagnosed for CAPS or sJIA would definitely further strengthen the clinical application.

## Conclusions

In summary, our data suggested that the rapid and excessive-production of IL-1β, IL-18 and caspase-1 in the LPS inflammasome stimulation assay on PBMC cultures can assist the differentiation of persistent sJIA and CAPS.

## Supplementary Information

Supplementary Information.

## Data Availability

All relevant data were displayed in the table and figures.

## Abbreviations

ATP: Adenosine triphosphate
CAPS: Cryopyrin-associated periodic syndrome
CIAS1: Cold-induced autoinflammatory syndrome 1
FCAS: Familial cold autoinflammatory syndrome
IFN: Interferon
IL: Interleukin
LPS: Lipopolysaccharide
MAS: Macrophage activation syndrome
MWS: Muckle–Wells syndrome
NF-κB: Nuclear factor kappa B
NLRP12: NOD-like receptor family pyrin domain containing 12
NLRP3: NOD-like receptor family pyrin domain containing 3
NOMID: Neonatal onset multisystem inflammatory disease
PBMCs: Peripheral blood mononuclear cells
PCR: Polymerase chain reaction
SAA: Serum amyloid A
sJIA: Systemic juvenile idiopathic arthritis
TLR: Toll like receptor
TNF-α: Tumor necrosis factor alpha
