# Supplementary material for: Lipopolysaccharide stimulation test on cultured PBMCs assists the discrimination of cryopyrin-associated periodic syndrome from systemic juvenile idiopathic arthritis
Source: Sci Rep. 2021 Jun 7;11:11903. doi: 10.1038/s41598-021-91354-5 (PMC8185076; doi:10.1038/s41598-021-91354-5)
Supplement: Supplementary file 1 — Supplementary Information. [file 41598_2021_91354_MOESM1_ESM.pdf]

Supplementary Table 1. Clinical manifestations and relevant comorbidities of the patients diagnosed with CAPS.

Patient No.	#1	#2	#3	#4	#5	#6	#7	#8	#9	#10	#11
Gender	F	F	M	F	F	M	F	F	F	F	F
Age of onset (yr)	8	<1	1	2	5	5	2	15	4	12	16
Family history	+	+	+	+	+	+	+	-	-	+	-
Genetic variants	NLRP3 A439V	NLRP3 A439V	NLRP3 A439V	NLRP3 A439V	NLRP3 A439V	NLRP3 V70M	NLRP3 E457D	-	-	-	-
Clinical phenotype	FCAS	WMS	WMS	WMS	FCAS	FCAS	FCAS	FCAS	FCAS	FCAS	FACS
Recurrent fever	-	+	+	+	-	+	-	+	+	+	+
Lymphadenopathy	-	+	+	-	-	-	-	-	-	-	-
Cold-induced urticarial like rash	+	+	+	+	+	+	+	+	+	+	+
Arthritis / arthralgia	+	+	+	+	+	-	+	+	+	+	+
Ocular manifestations	-	-	uveitis	-	-	-	-	-	-	-	conjunctivitis
CNS manifestations	-	sensorineural defects	aseptic meningitis	sensorineural hearing loss	chronic headache	-	-	-	-	-	-
Cardiovascular comorbidities	-	-	-	-	-	-	-	-	-	-	-
Hypertension	-	-	-	-	-	-	-	-	-	-	-
Hematuria	-	-	-	-	-	-	-	-	-	-	-
Proteinuria	-	-	-	-	-	-	-	-	-	-	-
Renal amyloidosis	NA	NA	NA	NA	NA	NA	NA	NA	NA	NA	NA
Smoking	-	-	-	-	-	-	-	-	-	-	-

Abbreviations: CAPS - Cryopyrin-Associated Periodic Syndrome; FCAS - familial cold autoinflammatory syndrome; WMS - Muckle–Wells syndrome; CNS – central nervous system; NA – not applicable
